# Predicting Contrast-Induced Nephropathy in NSTEMI: The Role of the HALP Score

**DOI:** 10.3390/medicina62010016

**Published:** 2025-12-22

**Authors:** Evliya Akdeniz, Yasin Yüksel, Cennet Yildiz, Bünyamin Aişeoğlu, Salih Gürkan Ergün, Fatma Nihan Turhan Çağlar, Dilay Karabulut

**Affiliations:** 1 Department of Cardiology, Bakırköy Dr. Sadi Konuk Training and Research Hospital, 34147 İstanbul, Turkey; cennet_yildiz@live.com (C.Y.); bunyaminaiseoglu@gmail.com (B.A.); grknergn@gmail.com (S.G.E.); nhnturhan@gmail.com (F.N.T.Ç.); dilay_karakozak@hotmail.com (D.K.); 2 Department of Cardiology, İSTÜN Beylikdüzü Kolan Hospital, 34528 İstanbul, Turkey; dryasinyuksel@gmail.com

**Keywords:** contrast-induced nephropathy, coronary angiography, HALP score, NSTEMI

## Abstract

*Background and Objectives:* Contrast-induced nephropathy (CIN) remains a significant complication following invasive coronary procedures. The HALP score—a composite index derived from hemoglobin, albumin, lymphocyte, and platelet counts—reflects nutritional and inflammatory status and may serve as a predictive biomarker for CIN. The aim of our study is to evaluate the relationship between the HALP score and the development of CIN in non-ST segment elevation myocardial infarction (NSTEMI) patients undergoing coronary angiography (CAG) or percutaneous coronary intervention (PCI). *Materials and Methods:* This retrospective study included 577 NSTEMI patients who underwent CAG or PCI between December 2022 and June 2025. Patients were divided into two groups based on CIN development. The HALP score was calculated and compared between groups. *Results:* Of the 577 NSTEMI patients included, 74 (12.8%) developed CIN. Patients who developed CIN were significantly older and had a higher prevalence of diabetes mellitus (DM), worse baseline renal function, and lower levels of hemoglobin, albumin, HDL cholesterol, and lymphocytes (*p* < 0.001). They also showed higher neutrophil counts, troponin-T levels, and received greater volumes of contrast media (CM). Oral antidiabetic drug (OAD) use was positively associated with CIN, while angiotensin-converting enzyme inhibitor/angiotensin II receptor blocker use showed a negative association in univariate analysis. The HALP score was significantly lower in the CIN (+) group than CIN (−) group (15.88 ± 28.48 vs. 53.86 ± 28.48, *p* < 0.001). Multivariate analysis identified older age, DM, reduced left ventricular ejection fraction, elevated creatinine, increased neutrophils, lower hemoglobin, albumin, and lymphocytes, and higher CM volume as independent predictors of CIN. The HALP score remained a strong inverse predictor of CIN (OR: 0.895; 95% CI: 0.865–0.924; *p* < 0.001) and the Mehran score was positively associated with CIN risk (OR: 1.578; 95% CI: 1.154–2.087; *p* < 0.001). Covariate-adjusted receiver operating characteristic (AROC) analysis demonstrated that the HALP score showed good predictive accuracy (AUC: 0.780), with 74.3% sensitivity and 83.3% specificity at a cutoff of 24.1. *Conclusions:* The HALP score is a simple, accessible, and cost-effective biomarker with strong predictive value for CIN in NSTEMI patients undergoing invasive coronary procedures.

## 1. Introduction

The prevalence of cardiovascular diseases continues to increase worldwide, and ischemic heart disease is responsible for nearly half of all cardiovascular deaths [1]. Among acute coronary syndromes, despite advances in diagnostic and therapeutic methods and the use of tailored, patient-specific approaches, non-ST elevation myocardial infarction (NSTEMI) has shown an increasing trend in incidence over time. In recent years, the prevalence of NSTEMI has risen significantly, to the extent that NSTEMI patients now constitute more than 60% of all acute myocardial infarction cases [2,3].

Coronary angiography (CAG) and percutaneous coronary intervention (PCI) are of critical importance in the contemporary standard of care for NSTEMI [4]. There is substantial and compelling evidence indicating that the implementation of an invasive strategy, including revascularization, is significantly associated with improved clinical outcomes in patients with NSTEMI [5,6].

Although the invasive strategy represents a highly valuable diagnostic and therapeutic approach in the management of NSTEMI, the potential complications associated with CAG and PCI should always be carefully considered and taken into account during the clinical decision-making process. The potential complications encompass a broad spectrum, including but not limited to local vascular complications, allergic reactions, infections, contrast-induced nephropathy (CIN), central nervous system events, myocardial infarction, conduction disturbances, and, in rare cases, death [7]. The rate of CIN following exposure to contrast media (CM) varies across different patient populations and clinical settings, ranging from approximately 10–13% and reaching up to as high as 28% in certain groups [8,9,10].

CIN has been shown to be associated with both increased in-hospital and long-term mortality, underscoring its clinical significance and highlighting its potential severity as a complication that may arise following CAG and PCI. This association serves as a critical reminder to clinicians that CIN is not only common but also potentially fatal [11,12]. Due to the undeniable negative impact of CIN on patient survival, several risk scoring systems have been developed to predict its occurrence, among which the Mehran risk score is one of the most widely recognized and commonly used [13].

The HALP score is a composite index calculated from routinely obtained blood parameters—hemoglobin, albumin, lymphocyte count, and platelet count—and serves as a surrogate marker reflecting the patient’s nutritional status and systemic inflammatory response. It has been increasingly utilized in clinical research as a prognostic indicator across various disease states [14,15,16].

In our study, we aimed to evaluate the association between the HALP score—derived from hemoglobin, albumin, lymphocyte count, and platelet count—and the development of CIN following CAG or PCI in patients diagnosed with NSTEMI.

## 2. Materials and Methods

### 2.1. Study Population

Between December 2022 and June 2025, consecutive patients who were hospitalized with a diagnosis of NSTEMI and underwent either CAG or PCI were retrospectively included in our study. The inclusion criteria were age > 18 years and to be undertaken to invasive strategy. Exclusion criteria included refusal to participate, the presence of end-stage renal disease (ESRD) requiring hemodialysis, known allergy to iodinated contrast agents, active infection or sepsis, recent or ongoing use of nephrotoxic drugs, recent exposure to CM or acute kidney injury at baseline.

Baseline demographic characteristics, along with clinical and laboratory variables, were collected from the hospital information system. The study cohort was divided into two groups based on the development of contrast-induced nephropathy: CIN (+) group and the CIN (−) group. Clinical variables associated with CIN were analyzed between the two groups, and independent predictors of CIN development were identified.

Written informed consent was obtained from all patients enrolled in the study, and the study was conducted in full accordance with the principles of the Declaration of Helsinki.

### 2.2. Definitions

CIN is typically defined as either an absolute increase in serum creatinine of ≥0.5 mg/dL or a relative increase of ≥25% from baseline, occurring within 48–72 h following exposure to iodinated CM [13].

ESRD is defined as the terminal and irreversible phase of chronic kidney disease, characterized by near-complete or complete loss of renal function, typically indicated by a sustained reduction in glomerular filtration rate (GFR) below 15 mL/min/1.73 m^2^ or the need for renal replacement therapy [17].

NSTEMI is defined as an acute myocardial infarction characterized by recent ischemic symptoms or signs, a rise and/or fall in cardiac troponin indicating acute myocardial injury, and the absence of persistent ST-segment elevation on the 12-lead electrocardiography (ECG), although other ECG abnormalities such as ST-segment depression or T-wave alterations may be present [3].

The HALP score is defined as a surrogate marker reflecting nutritional status, immune function, and levels of systemic inflammation, and is calculated by multiplying hemoglobin, albumin, and lymphocyte counts, then dividing the result by the platelet count [14].

### 2.3. Statistical Analysis

Continuous variables were presented as mean ± standard deviation for normally distributed data, while variables that were non-normally distributed were expressed as median values with interquartile ranges. Categorical variables were presented as frequencies and percentages. Comparisons between patients with and without CIN were conducted using the independent Student’s t-test for normally distributed continuous variables and the Mann–Whitney U test for non-normally distributed variables. Comparative analysis of categorical variables was performed using the Chi-square test. To evaluate and compare the discriminative performance of the HALP and Mehran scores, covariate-adjusted receiver operating characteristic (AROC) analyses were conducted, adjusting for variables that reached statistical significance in the multivariable analysis. For the HALP score, adjustments were made for age, diabetes mellitus (DM), baseline creatinine, CM volume and left ventricular ejection fraction (LVEF), as these variables may influence the risk of CIN. In contrast, for the Mehran score, variables such as age, renal function, DM, anemia, and contrast volume were not reintroduced as covariates because they are intrinsic components of the score itself. Therefore, adjustment for the Mehran score was restricted to LVEF, in order to account for potential external confounding factors. Pairwise comparisons of the areas under the ROC curves (AUCs) were performed using the DeLong method.

Univariable logistic regression analysis was used to identify potential predictors of CIN, and variables that reached statistical significance in the univariable analysis were subsequently included in a multivariable logistic regression model to determine independent predictors. Due to correlations between creatinine and GFR, DM and oral antidiabetic drug (OAD) use, as well as creatinine and intravenous hydration, only one variable from each correlated pair was included in the multivariable analysis. A two-tailed *p* value of <0.05 was considered statistically significant.

All statistical analyses were performed using SPSS software for Windows, version 25.0 (IBM Corporation, Armonk, NY, USA) and R software, version 4.5.0 (R Foundation for Statistical Computing, Vienna, Austria).

### 2.4. Endpoint

The primary endpoint of our study was contrast-induced acute kidney injury.

## 3. Results

A total of 577 patients were enrolled in the study, including 74 individuals (12.8%) who developed CIN. Patients who developed CIN tended to be significantly older than those in the CIN (−) group (median 71.00 vs. 61.00 years, *p* < 0.001). The proportion of female patients was comparable between the two groups. Chronic conditions, particularly DM, were more frequently observed among patients who developed CIN (52.7% vs. 30.2%, *p* < 0.001), suggesting a stronger association with this comorbidity. In contrast, the prevalence of hypertension (HTN) and hyperlipidemia did not differ significantly. The use of angiotensin-converting enzyme inhibitors (ACEIs) or angiotensin II receptor blockers (ARBs) was significantly lower in the CIN (+) group compared to the CIN (–) group (62.2% vs. 83.1%, *p* < 0.001). Conversely, OAD use was more prevalent in patients who developed CIN (52.7% vs. 30.0%, *p* < 0.001). Although statin use was slightly higher in the CIN (+) group, the difference was not statistically significant (*p* = 0.344). Patients who developed CIN had notably worse kidney function at baseline, with higher serum creatinine levels (1.29 ± 0.48 mg/dL vs. 0.80 ± 0.42 mg/dL, *p* < 0.001) and lower estimated GFR (46.00 vs. 94.00 mL/min/1.73 m^2^, *p* < 0.001). CIN (+) patients also received higher volumes of CM (median 219.23 mL vs. 184.61 mL, *p* = 0.018) and more frequently underwent preprocedural hydration (83.8% vs. 7.8%, *p* < 0.001). Compared to patients in CIN (−) group, CIN (+) patients had significantly lower levels of hemoglobin, albumin, HDL cholesterol and lymphocyte count as well as lower LVEF. Conversely, neutrophil count and troponin-T were higher in CIN (+) patients than those in CIN (−) group. Notably, the HALP score was significantly lower in patients who developed CIN compared to those who did not (15.88 vs. 53.86, *p* < 0.001). Similarly, the Mehran score was significantly higher in patients with CIN (10.34 ± 4.58) compared to those without CIN (5.87 ± 3.94), (*p* < 0.001). Baseline characteristics, clinical, and laboratory variables are presented in Table 1.

Univariable logistic regression analysis identified several variables associated with the development of CIN (Table 2). Patients with older age and those with DM were more likely to develop CIN. Lower LVEF was associated with CIN. Baseline renal function markers showed strong associations, with baseline creatinine increasing risk (OR: 8.330, 95% CI: 4.559–15.221, *p* < 0.001) and baseline GFR being protective (OR: 0.923, 95% CI: 0.908–0.938, *p* < 0.001), as was 24–48 h GFR (OR: 0.867, 95% CI: 0.839–0.898, *p* < 0.001). Higher CM volume (OR: 1.004, 95% CI: 1.00–1.007, *p* = 0.007) and preprocedural hydration (OR: 1.510, 95% CI: 1.408–1.619, *p* < 0.001) were also significantly associated with CIN. The use of OAD was significantly associated with an increased risk of CIN (OR: 2.598, 95% CI: 1.584–4.259, *p* < 0.001), whereas the administration of ACEIs or ARBs had an inverse relationship with the development of CIN (OR: 0.334, 95% CI: 0.198–0.564, *p* < 0.001). Additionally, patients with lower levels of albumin, hemoglobin, and lower lymphocyte counts, as well as higher neutrophil counts, were more likely to develop CIN. The HALP score was markedly reduced in patients who developed CIN (OR: 0.952; 95% CI: 0.939–0.965; *p* < 0.001), and the Mehran score was also significantly associated with CIN risk (OR: 1.643; 95% CI: 1.262–2.187; *p* < 0.001), suggesting that both scores might serve as predictors for the development of CIN.

Due to multicollinearity among selected variables—including baseline creatinine and GFR, DM and OAD use, as well as creatinine and intravenous hydration—only one variable from each correlated pair was included in the multivariable logistic regression analysis. Accordingly, multivariable logistic regression analysis identified several independent predictors of CIN. Independent predictors of CIN included advanced age and the presence of DM, both of which significantly increased the risk. Additionally, reduced LVEF, elevated baseline creatinine levels, increased neutrophil counts, and higher volumes of CM were consistently associated with CIN. Lower levels of hemoglobin and albumin and lower lymphocyte counts also emerged as significant predictors, underscoring the role of nutritional and inflammatory status in CIN development. In contrast, the use of ACEI/ARB was not significantly associated with the risk of CIN. Furthermore, the HALP score, serving as a surrogate marker of nutritional and inflammatory status, demonstrated a strong inverse association with the risk of CIN (OR: 0.895, 95% CI: 0.865–0.924, *p* < 0.001). In comparison, the Mehran score was positively associated with CIN risk (OR: 1.578; 95% CI: 1.154–2.087; *p* < 0.001), indicating that both scores independently predicted the likelihood of developing CIN. Detailed results of the multivariate analysis are presented in Table 3.

**Figure 1:**
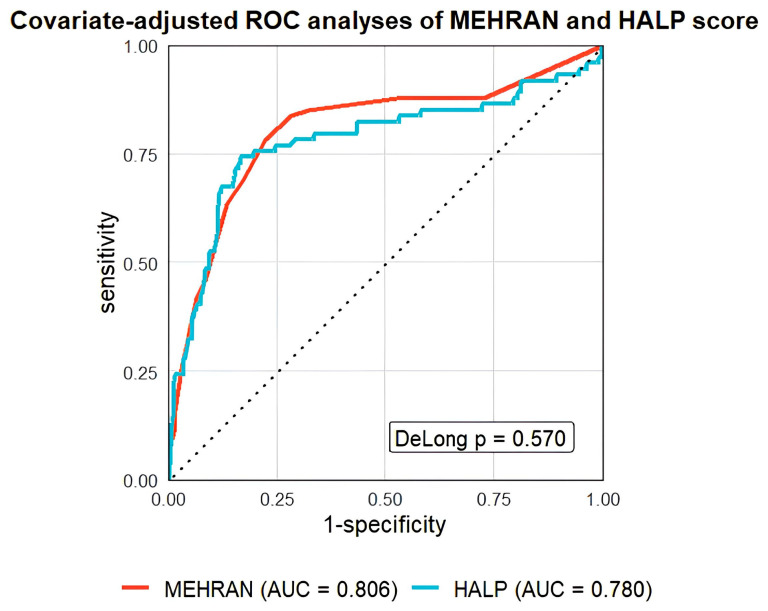
Adjusted Receiver Operating Characteristic (AROC) analysis of HALP and Mehran scores for CIN. AUC: Area Under Curve, CIN: Contrast-Induced Nephropathy, HALP: Hemoglobin, Albumin, Lymphocyte, and Platelet score.

AROC analysis was performed to evaluate the predictive performance of both HALP and Mehran scores for the development of CIN (Figure 1). The HALP score exhibited good discriminatory ability for predicting CIN, with an area under the curve (AUC) of 0.780 (95% CI: 0.709–0.851, *p* < 0.001); at an optimal cutoff value of 24.1, the HALP score achieved a sensitivity of 74.3% and a specificity of 83.3%. The AUC for the Mehran score was 0.806 (95% CI: 0.743–0.869, *p* < 0.001). Using a cutoff value of 7.5, the Mehran score demonstrated a sensitivity of 78.3% and a specificity of 77.7% for predicting CIN. The difference between the two AUCs was −0.029, with a 95% confidence interval ranging from −0.116 to 0.064. Comparison of the two ROC curves using the DeLong method indicated that this difference was not statistically significant (*p* = 0.570), suggesting that the HALP and Mehran scores have comparable discriminative performance for predicting CIN (Table 4).

## 4. Discussion

This study aimed to examine the relationship between the HALP score—a composite indicator encompassing nutritional and immunological status—and the occurrence of CIN in patients with NSTEMI undergoing CAG and/or PCI. Our results revealed that patients with lower HALP scores faced a significantly higher risk of developing CIN, highlighting the important role that compromised nutritional and immune health may play in increasing vulnerability to this complication.

Based on the comparable AUCs of the HALP and Mehran scores, it can be inferred that while the Mehran score is calculated using multiple clinical parameters, the HALP score relies on only four laboratory parameters, yet it demonstrates a similar predictive performance for CIN in NSTEMI patients. This suggests that the HALP score may offer a more practical and rapid tool, providing clinicians with valuable information for decision-making.

Cardiovascular diseases remain the leading cause of mortality worldwide to this day [1]. CAG and PCI are pivotal diagnostic and therapeutic tools that play a central role in the diagnosis and treatment of cardiovascular diseases. Given the frequent use of invasive procedures, complications associated with these interventions are not uncommon in contemporary clinical practice. One of these complications is CIN. Although the incidence of CIN following CM exposure varies across different clinical studies in cardiology practice, meta-analyses conducted in this field have estimated this rate to be approximately 10–13% [8,9]. In our study, the incidence of CIN in patients diagnosed with NSTEMI undergoing CAG or PCI was determined to be 12.8%, which is consistent with the rates reported in the literature.

Although the exact mechanisms underlying the CIN occurrence remain unclear, several potential pathways have been proposed. Firstly, CM induces vasoconstriction within the renal vasculature, resulting in an imbalance between renal oxygen supply and demand [18,19]. Secondly, the direct tubular toxicity of CM represents another key mechanism contributing to the pathophysiology of CIN. CM can induce cytotoxic effects on both renal tubular cells and vascular endothelial cells, leading to necrosis and apoptosis, which contribute to the development of CIN [20]. The third potential mechanism involves contrast media-induced generation of reactive oxygen species (ROS), which contribute to oxidative stress and subsequent renal injury [21].

Beyond the direct cytotoxic effects and indirect hemodynamic alterations, inflammation represents one of the key pathological mechanisms in the development of CIN. Notably, a meta-analysis by Wu et al. highlighted a significant association between elevated pre-procedural C-reactive protein levels and the subsequent development of CIN in patients undergoing coronary intervention [22]. Moreover, the PRATO-ACS (Protective Effect of Rosuvastatin and Antiplatelet Therapy on Contrast-Induced Nephropathy and Myocardial Damage in Patients With Acute Coronary Syndrome Undergoing Coronary Intervention) study demonstrated a significant association between the development of CIN and elevated levels of high-sensitivity C-reactive protein (hs-CRP) [23]. These findings underscore the contributory role of systemic inflammation in the pathophysiological process of CIN.

In addition to the aforementioned mechanisms, there is a significant link between nutritional status and the development of CIN. Poor nutritional status has been shown to increase renal vulnerability to injury, potentially exacerbating the risk of CM associated renal injury [24]. As a surrogate marker of nutritional status, albumin demonstrated a significant association with the development of CIN, with hypoalbuminemia identified as a risk factor for CIN [25].

The HALP score, a composite index reflecting nutritional and inflammatory status, was first introduced by Chen et al. as a novel prognostic marker combining hemoglobin, albumin, lymphocyte, and platelet counts to assess nutritional and inflammatory status, particularly in gastric carcinoma patients [14]. In addition to the direct and indirect effects of CM, inflammatory responses and nutritional status represent critical pathophysiological mechanisms contributing to the development of CIN. Consequently, the statistically significant association identified between the HALP score and CIN incidence in our study is consistent with and supported by extant scientific evidence.

In this context, the HALP score presents considerable advantages for predicting the development of CIN in patients undergoing coronary interventions. Primarily, it is a straightforward and practical metric that can be readily derived from routinely available laboratory parameters. Its simplicity, combined with cost-effectiveness, renders it an accessible and efficient tool for risk stratification in routine clinical settings. Moreover, the HALP score offers clinicians timely and meaningful insights into a patient’s nutritional and inflammatory status, thereby facilitating more informed and proactive clinical decision-making. This dual utility underscores its potential value as both a prognostic marker and a guide for comprehensive patient management.

### 4.1. Study Novelty

Although established risk factors for CIN—such as advanced age, DM, impaired renal function, and CM volume—are already well recognized, they were intentionally re-examined in this study to provide clinical context for assessing the added value of the HALP score. Our findings show that the HALP score remains a strong and independent inverse predictor of CIN even after accounting for these traditional risk factors, indicating that it captures additional pathophysiological information not fully reflected by conventional variables alone. By combining routinely available laboratory parameters into a single, easy-to-use index, the HALP score emerges as a practical and cost-effective tool for early CIN risk stratification that complements, rather than replaces, existing risk assessment models. Taken together, these findings support the inclusion of the HALP score as a meaningful prognostic biomarker in the management of patients undergoing contrast exposure. To our knowledge, this is the first study to evaluate the HALP score in the context of CIN among NSTEMI patients undergoing coronary interventions. Recognizing the interplay between nutritional status, inflammation, and renal vulnerability opens new avenues for targeted interventions and improved patient outcomes.

### 4.2. Study Limitations

While our study provides valuable insights into the relationship between the HALP score and CIN, several limitations warrant consideration. As a single-center observational study, the findings may not be fully generalizable to diverse patient populations or different healthcare settings. The relatively modest sample size, especially in the group developing CIN, may limit the robustness of some statistical associations. Moreover, despite the promising predictive value of the HALP score, unmeasured factors such as variations in hydration protocols, contrast media types, and other underlying comorbidities could have influenced the outcomes. Finally, the observed between the HALP score and CIN should be interpreted with caution and merits further in-depth evaluation. Since the HALP score was originally developed and validated in oncological settings, its application to CIN represents an extension beyond its initial clinical purpose and therefore requires careful consideration. The retrospective nature of the present analysis further limits causal inference. Future research, ideally in the form of well-designed prospective studies or analyses focusing on patients with more severe forms of CIN, would be valuable in strengthening the validity and clinical relevance of these findings and in clarifying the potential role of HALP in this context.

## 5. Conclusions

In summary, our findings underscore the valuable role of the HALP score as a simple, accessible and cost-effective biomarker that integrates nutritional and inflammatory status to help predict the risk of CIN in patients undergoing coronary interventions. By providing timely insight into patients’ underlying health conditions, the HALP score can empower clinicians to identify those at greater risk and tailor preventive measures accordingly. While our results are promising, further research is warranted to confirm applicability of HALP score in routine clinical practice. Ultimately, the HALP score holds potential to improve patient outcomes by enhancing early detection and intervention in this vulnerable group.

## Figures and Tables

**Table 1:** Comparison of Demographic, Clinical, and Laboratory Parameters Between Overall Cohort, CIN (+) and CIN (−) Groups.

Variable	Overall Cohort (%)	CIN (+) Group (n = 74)	CIN (−) Group (n = 503)	*p*
Age (years)	62.75 ± 12.23	71.00 (62.00–81.25)	61.00 (53.00–70.00)	<0.001
Female gender	155 (26.9)	24 (32.4)	131 (26)	0.255
Smoking	344 (59.6)	50 (67.5)	294 (58.5)	0.223
DM	191 (33.1)	39 (52.7)	152 (30.2)	<0.001
HTN	524 (90.8)	69 (93.2)	455 (90.5)	0.421
HL	492 (85.3)	64 (86.5)	428 (85.1)	0.749
LVEF (%)	54.40 ± 9.11	50.00 (40.00–55.00)	60.00 (50.00–60.00)	<0.001
ACEI/ARB	464 (80.4)	46 (62.2)	418 (83.1)	<0.001
OAD	190 (32.9)	39 (52.7)	151(30)	<0.001
Statin	236 (40.9)	34 (45.9)	202 (40.2)	0.344
CM volume (mL)	186.92 (147.69–247.30)	219.23 (158.19–281.80)	184.61 (147.69–240.92)	0.018
Preprocedural hydration	101 (17.5)	62 (83.8)	39 (7.8)	<0.001
Admission glucose (mg/dL)	168.02 ± 102.83	150.00 (106.00–200.00)	144.00 (110.00–188.25)	0.843
Baseline creatinine(mg/dL)	0.94 ± 0.48	1.29 (0.99–1.85)	0.80 (0.70–0.94)	<0.001
Baseline GFR (mL/min/1.73 m^2^)	86.04 ± 24.34	46.00 (32.05–68.00)	94.00 (79.00–105.00)	<0.001
24–48 h GFR (mL/min/1.73 m^2^)	80.00 (60.00–96.00)	27.00 (15.62–41.25)	85.00 (67.00–98.00)	<0.001
LDL (mg/dL)	125.65 ± 42.10	121.50 (91.50–137.50)	127.00 (94.00–155.00)	0.125
HDL (mg/dL)	42.20 ± 10.89	35.00 (31.00–41.25)	42.00 (36.00–48.00)	<0.001
Albumin (gr/dL)	4.06 ± 0.47	3.6(3.3–4.7)	4.2 (3.9–4.4)	<0.001
Hemoglobin (g(dL)	13.14 ± 1.99	10.90 (9.80–12.30)	13.70 (12.30–14.80)	<0.001
Neutrophil (10^9^/L)	5.95 ± 2.82	7.18(4.68–9.58)	5.40 (4.20–6.71)	<0.001
Platelet (10^9^/L)	253.92 ± 79.22	255.00 (211.25–325.00)	242.00 (200.00–291.00)	0.078
Lymphocyte (10^9^/L)	2.23 ± 0.89	1.01 (0.77–1.64)	2.31 (1.80–2.85)	<0.001
Troponin-T (ng/L)	4386.80 ± 9906.54	1884.50 (520.25–9179.75)	504.00 (122.00–3323.00)	<0.001
HALP score	52.20 ± 28.48	15.88 (11.17–21.55)	53.86 (38.22–74.54)	<0.001
Mehran score	5.87 ± 3.94	10.34 ± 4.58	5.20 ± 3.37	<0.001

ACEI: Angiotensin-Converting Enzyme Inhibitor; ARB: Angiotensin II Receptor Blocker; CIN: Contrast-Induced Nephropathy; CM: Contrast Media; DM: Diabetes Mellitus; GFR: Glomerular Filtration Rate; HALP: Hemoglobin, Albumin, Lymphocyte, and Platelet score; HDL: High-Density Lipoprotein; HL: Hyperlipidemia; HTN: Hypertension; LDL: Low-Density Lipoprotein; LVEF: Left Ventricular Ejection Fraction; OAD: Oral Antidiabetic Drug.

**Table 2:** Univariate Logistic Regression Analysis for Predictors of CIN.

Variable	Odds Ratio (OR)	95% Confidence Interval (CI)	*p*
Age	1.067	1.045–1.091	<0.001
DM	2.573	1.569–4.219	<0.001
LVEF	0.917	0.895–0.939	<0.001
OAD	2.598	1.584–4.259	<0.001
ACEI/ARB	0.334	0.198–0.564	<0.001
Baseline creatinine	8.330	4.559–15.221	<0.001
Baseline GFR	0.923	0.908–0.938	<0.001
24–48 h GFR (mL/min/1.73 m^2^)	0.867	0.839–0.898	<0.001
CM volume	1.004	1.001–1.007	0.007
Preprocedural hydration	1.510	1.408–1.619	<0.001
HDL	0.924	0.894–1.001	0.054
Albumin	0.838	0.797–0.881	<0.001
Hemoglobin	0.941	0.927–0.955	<0.001
Neutrophil	1.231	1.116–1.359	<0.001
Lymphocyte	0.794	0.755–0.836	<0.001
Troponin-T	1.000	1.000–1.000	0.052
HALP score	0.952	0.939–0.965	<0.001
Mehran score	1.643	1.262–2.187	<0.001

Abbreviations as Table 1.

**Table 3:** Multivariate Logistic Regression Analysis for Predictors of CIN.

Variable	Odds Ratio (OR)	95% Confidence Interval (CI)	*p*
**MODEL A**			
Age	1.038	1.005–1.071	0.023
DM	2.956	1.417–6.167	0.004
LVEF	0.924	0.895–0.953	<0.001
ACEI/ARB	0.670	0.296–1.516	0.336
Baseline creatinine	4.028	2.302–7.047	<0.001
Albumin	0.920	0.854–0.990	0.027
Hemoglobin	0.966	0.946–0.986	0.001
Neutrophil	1.164	1.058–1.281	0.002
Lymphocyte	0.926	0.883–0.972	0.002
CM volume	1.006	1.002–1.010	0.007
**MODEL B**			
Age	1.043	1.007–1.080	0.020
DM	3.108	1.373–7.033	0.007
LVEF	0.922	0.889–0.956	<0.001
ACEI/ARB	0.742	0.301–1.832	0.518
Baseline creatinine	4.981	2.385–10.404	<0.001
Neutrophil	1.104	1.012–1.204	0.026
CM volume	1.005	1.001–1.009	0.031
HALP score	0.895	0.865–0.924	<0.001
**MODEL C**			
LVEF	0.955	0.928–0.983	0.002
ACEI/ARB	0.433	0.222–1.084	0.055
Albumin	0.931	0.875–0.989	0.021
Neutrophil	1.171	1.074–1.277	<0.001
Lymphocyte	0.893	0.853–0.935	<0.001
Mehran score	1.578	1.154–2.087	<0.001

Abbreviations as Table 1.

**Table 4:** Comparison of HALP and Mehran Scores for Predicting CIN Using AROC Analysis.

	AUC	*p*	95% CI	Cutoff	Sensitivity (%)	Specificity (%)
HALP score	0.780	<0.001	0.709–0.851	24.1	74.3	83.3
Mehran score	0.806	<0.001	0.743–0.869	7.5	78.3	77.7
AUC difference (HALP vs. Mehran): −0.029 (95% CI: −0.116 to 0.064), *p* = 0.570 (DeLong test)						

AROC: Adjusted Receiver Operating Characteristic, AUC: Area Under Curve, CI: Confidence Interval, CIN: Contrast-Induced Nephropathy, HALP: Hemoglobin, Albumin, Lymphocyte, and Platelet score.

## Data Availability

Access to the raw data is restricted for privacy and ethical reasons.
